# Should C-reactive protein concentration at ICU discharge be used as a prognostic marker?

**DOI:** 10.1186/1471-2253-10-17

**Published:** 2010-09-27

**Authors:** Joana Silvestre, Luís Coelho, Pedro Póvoa

**Affiliations:** 1 Polyvalent Intensive Care Unit, São Francisco Xavier Hospital, CEDOC, Faculty of Medical Sciences, New University of Lisbon, Hospital de São Francisco Xavier, Centro Hospitalar de Lisboa Ocidental, Estrada do Forte do Alto do Duque, 1449-005 Lisboa, Portugal

## Abstract

**Background:**

About one third of hospital mortality in critically ill patients occurs after Intensive Care Unit (ICU) discharge. Some authors have recently hypothesized that unresolved or latent inflammation and sepsis may be an important factor that contributes to death following successful discharge from the ICU.

**Aim:**

The aim of our study was to determine the ability of the clinical and inflammatory markers at ICU discharge to predict post-ICU mortality.

**Methods:**

A prospective observational cohort study was conducted during a 14-month period in an 8 bed polyvalent ICU. Acute Physiology and Chronic Health Evaluation (APACHE) II score, Simplified Acute Physiology Score (SAPS) II, Sequential Organ Failure Assessment (SOFA) score, Therapeutic Intervention Scoring System-28 (TISS-28), C-reactive protein (CRP), white cell count (WCC) and body temperature of the day of ICU discharge were collected from patients who survived their first ICU admission.

**Results:**

During this period 156 patients were discharged alive from the ICU. A total of 29 patients (18.6%) died after ICU discharge. There were no differences in clinical and demographic characteristics between survivors and nonsurvivors. C-reactive protein levels at ICU discharge were not significantly different between survivors and nonsurvivors. The area under receiver operating characteristics curves of APACHE II, SAPS II, SOFA, TISS-28, CRP, WCC and body temperature at ICU discharge as prognostic markers of hospital death were 0.76 (95% confidence interval (CI) 0.67-0.86); 0.75 (95% CI 0.66-0.85); 0.72 (95% CI 0.62-0.83); 0.64 (95% CI 0.52-0.77); 0.55 (95% CI 0.43-0.67); 0.55 (95% CI 0.42-0.66) and 0.54 (95% CI 0.44-0.67) respectively. The hospital mortality rate of the patients with CRP <5, 5-10, >10 mg/dL was 15.1%, 16.1% and 33.3% respectively (p = NS).

**Conclusions:**

At ICU discharge serum CRP concentration was a poor marker of post-ICU prognosis. Post-ICU death appears to be unrelated to the persistent inflammatory response.

## Background

Critically ill patients are responsible for 10-25% global hospital costs [1]. The ability to identify critically ill patients who will not survive until hospital discharge may allow identification of high risk patients leading to more conservative strategies of ICU discharge.

About one third of hospital mortality of critically ill patients occurs after Intensive Care Unit (ICU) discharge [2].

Smith et al. observed in 283 patients discharged from the ICU to hospital wards that patients with higher Therapeutic Intervention Scoring System (TISS)-28 had higher post-ICU mortality (TISS-28 >20 = 21.4% vs. TISS-28 <10 = 3.7%, p < 0,0001) [3]. Several other risk-prediction models have been used to predict in-hospital mortality after patient discharge from the ICU [4,5]. However risk estimated by these models showed considerable variation across the disease spectrum of ICU patients.

Post ICU deaths arise mainly as a result of incomplete resolution of the primary condition or from the development of new complications [6-8]. Some authors have recently hypothesized that unresolved or latent inflammation and sepsis may be an important factor that contributes to death following successful discharge from the ICU [6].

In two recent studies with critically ill patients, a high CRP concentration at ICU discharge was associated with a subsequent increase in in-hospital mortality [6,9].

The aim of our study was to determine the ability of the clinical and inflammatory markers at ICU discharge to predict post ICU mortality.

## Methods

This study was a prospective, single center, observational study conducted during a 14-month period in the ICU of Garcia de Orta Hospital, an 8-bed multidisciplinary ICU.

The Hospital Ethics Committee approved the study design, and informed consent was waived because this was an observational study without any deviation from the current medical practice.

Patients were included in the study if they were discharged alive from the ICU and if they were more than 17 years old. Discharged criteria were clinical improvement without need of further organ support and/or intensive monitoring. Patients were followed until hospital death or hospital discharge. Only the first ICU admission was included.

The clinical predictors analyzed included Acute Physiology and Chronic Health Evaluation (APACHE) II [10], Simplified Acute Physiology Score (SAPS) II [11], Sequential Organ Failure Assessment scores (SOFA) [12] and Therapeutic Intervention Scoring System-28 (TISS-28) [13].

C-reactive protein, body temperature and white cells count (WCC) were measured at admission and then daily until discharge.

Measurement of CRP was performed by an immunoturbidimetric method (Tina-quant CRP; Roche Diagnostics, Mannheim, Germany).

We compared the clinical and laboratory data of survivors and nonsurvivors after ICU discharge.

A subgroup analysis between infected and non-infected patients was performed. Surgical and medical patients were also analyzed. We considered that the patient belong to the surgical group if the main reason of ICU admission was surgical, obstetric or trauma.

### Statistical Analyses

The outcome measure was post-ICU mortality. Continuous variables are presented as mean +/- standard deviation (SD), unless stated otherwise. Differences in continuous variables were performed with the parametric unpaired Student's *t *test and one-way ANOVA or with the nonparametric Mann-Whitney *U*-test or Kruskal-Wallis *H*-test according to data distribution. The Chi-square test was used to carry out comparisons between categorical variables.

C-reactive protein levels were categorized in three groups (CRP <5, 5-10, >10 mg/dL) and compared with mortality rate. Linear regression analysis was used to compare SOFA with CRP levels. Discrimination of APACHE II, SAPS II, SOFA, TISS-28, CRP, body temperature and WCC was tested to produce receiver-operating characteristic (ROC) curves. Areas under curves (AUC), with 95% confidence intervals (CI) were calculated in prediction of ICU mortality. A p value below 0.05 was considered statistically significant. Statistical analyses were performed using SPSS 16.0 software.

## Results

During a 14-month period a total of 262 patients were admitted in the ICU. The overall ICU mortality was 40%.

One hundred and fifty six patients were discharged alive from ICU to ward with a mean age of 55 ± 18 and 93 (60%) were males (Table 1).

**Table 1:** Baseline characteristics of the patients discharged from the Intensive care Unit

	All (n = 156)	Survivors (n = 127)	Nonsurvivors (n = 29)	*p *values
Age, yrs	55 ± 18	53 ± 19	62 ± 12	NS
Sex (M/F)	93/63	79/48	14/15	NS
APACHE II	14.6 ± 6.2	13.4 ± 5.3	20.0 ± 7.1	<0.001
SAPS II	28.6 ± 12.7	25.9 ± 9.6	40.5 ± 17.5	<0.001
TISS-28	25.1 ± 5.3	24.2 ± 4.3	28.8 ± 7.1	<0.001
SOFA	3.5 ± 2.7	3.0 ± 1.8	5.6 ± 4.7	<0.001
CRP (mg/dL)	8.5 ± 8.3	8.1 ± 8.0	10.1 ± 9.5	NS
Temperature (ºC)	36.9 ± 2.7	36.8 ± 3.0	37.2 ± 0.8	NS
WCC (x1000)/mL	11.3 ± 7.4	10.6 ± 6.0	14.4 ± 11.4	NS
Admission diagnosis (N)				
Respiratory	47	35	12	
Trauma	24	21	3	
Surgical	21	17	4	
Cardiovascular	21	17	4	
Neurological	14	13	1	
Obstetrics	6	6	0	
Gastroenterological	6	5	1	
Others	17	13	4	

Values expressed as mean ± standard deviation

A total of 29 patients (18.6%) died in hospital after ICU discharge. Clinical and demographic characteristics of post-ICU survivors and nonsurvivors are presented in table 1. The mean duration of follow-up post-ICU discharge was 34.8 days, with no difference between survivors and nonsurvivors (34.3 ± 26.8 versus 37.6 ± 24.9 days; *p *= NS). Nonsurvivors were sicker with higher levels of APACHE II, SAPS II, SOFA and TISS-28. (Table 1).

C-reactive protein was determined in all patients at ICU discharge. C-reactive protein values varied from 0.15 to 43.5 mg/dL.

Although 25% higher in nonsurvivors, CRP levels at ICU discharge was not significantly difference in relation to survivors (survivors - 8.1 ± 8.0 vs. nonsurvivors - 10.2 ± 12.0 mg/dL; *p *= NS). In addition, no correlation could be found between higher CRP levels and mortality. Post-ICU mortality rate of the patients with CRP <5, between 5-10, >10 mg/dL was 15.1% (N = 9), 16.1% (N = 9) and 33.3% (n = 11) respectively (p = NS).

**Figure 1:**
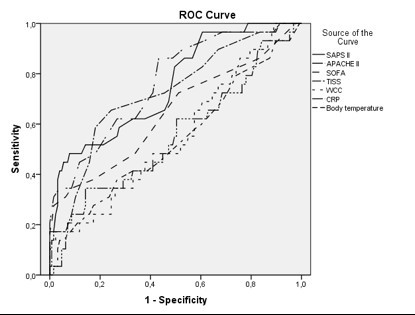
**Receiver operating characteristics (ROC) curves of Simplified Acute Physiology Score (SAPS) II, Acute Physiology and Chronic Health Evaluation (APACHE) II, Therapeutic Intervention Scoring System-28 (TISS-28), Sequential Organ Failure Assessment (SOFA) scores, serum C- reactive protein (CRP), body temperature and white cell count (WCC)**.

The area under the ROC curves of APACHE II, SAPS II, TISS-28, SOFA, CRP, WCC and body temperature and at ICU discharge as prognostic markers of post-ICU death were 0.76 (95% CI 0.67-0.86), 0.75 (95% CI 0.67-0.86), 0.72 (95% CI 0.62-0.83), 0.64 (95% CI 0.52-0.77), 0.55 (95% CI 0.43-0.68), 0.56 (95% CI 0.44-0.67) and 0.54 (95% CI 0.44-0.67) respectively (Figure 1).

### CRP as a prognostic marker in patients with previous documented infection

One hundred and thirty six out of 262 of the patients discharged from ICU presented at least one documented infection during their ICU stay. The mean age was 55 ± 17 years and 83 (61%) were males.

The primary reason for ICU admission was respiratory failure due to pneumonia. The in-hospital mortality rate from the patients with documented infection was 21%.

At ICU discharge, CRP values varied from 0.15 to 43.5 mg/dL (median 5.7 mg/dL). No differences in CRP levels were observed between survivors and nonsurvivors (8.4 ± 8.2 vs. 10.3 ± 9.6 mg/dL, p = NS, respectively) after discharge. However, the SAPS II, APACHE II, TISS-28 and SOFA score were significantly higher in nonsurvivors (Table 2).

**Table 2:** Baseline characteristics of the patients with documented infection

	Survivors (n = 127)	Nonsurvivors (n = 29)	*p *values
Age, yrs	54 ± 18	62 ± 12	*p *= NS
Sex (M/F)	69/39	14/14	*p *= NS
APACHE II	13.7 ± 4.9	20.3 ± 10.0	*p *< 0.001
SAPS II	26.2 ± 8.8	41.3 ± 17.2	*p *< 0.001
TISS-28	24.1 ± 3.6	29.1 ± 7.1	*p *< 0.001
SOFA	3.0 ± 1.7	5.7 ± 4.7	*p *< 0.001
CRP (mg/dL)	8.4 ± 8.2	10.3 ± 9.6	*p *= NS

Values expressed as mean ± standard deviation

The AUC showed a good discriminative power of post-ICU mortality for TISS-28 (AUC 0.75; 95% CI 0.665-0.86), SAPS II (AUC 0. 77; 95% CI 0.68-0.87) and APACHE II score (AUC 0.78; 95% CI 0.68-0.87). For CRP levels the AUC did not demonstrate a good discriminative power of post-ICU mortality (AUC 0.55; 95% CI 0.42-0.67).

### CRP as a prognostic marker in surgical and medical patients

In 51 patients the main admission diagnosis was surgical. The mean age was 49 ± 19 years and 33 (65%) were male.

The post-ICU mortality rate was 14% and no differences were observed between surgical and medical patients.

C-reactive protein levels at ICU discharge were significantly higher than in medical patients (6.5 ± 7.0 vs. 12.5 ± 9.2; p < 0.001). However no differences in CRP levels at ICU discharge could be found between survivors and nonsurvivors in both surgical and medical patients (5.9 ± 6.7 mg/dL vs. 8.7 ± 8.9 mg/dL; *p *= NS and 12.2 ± 8.6 mg/dL vs. 14.6 ± 13.0; *p *= NS, respectively).

CRP also did not demonstrate a good discriminative power of post-ICU mortality (Medical Group: AUC 0. 51; 95% CI 0.19-0.82 and Surgical Group: AUC 0.38; 95% CI 0.24-0.52).

## Discussion

In this prospective observational study with 156 patients discharged alive from ICU, we evaluated the relation between CRP levels at ICU discharge and post-ICU mortality. Our data demonstrate that CRP at ICU discharge was not correlated with in-hospital mortality. Even in patients with higher levels of CRP (>10 mg/dL) there was no significant increase in post-ICU mortality. In the subgroup analysis these data were similar and no association could be found between CRP levels and post-ICU mortality in patients with previous documented infection and in medical and surgical patients.

In a heterogeneous ICU patient population, Lobo et al. showed that admission CRP levels correlated with an increased risk of organ failure and death [14]. In addition, our group showed that persistently elevated CRP concentrations in infected critically ill patients were associated with poor outcome [15,16]. Recently, it has been described that in survivors of an acute infection could present a state of persistent inflammation that may lead to deterioration of other diseases, such as cardiovascular disease, and an increased long-term mortality [7].

Long-term mortality has been assessed in a recent multicenter study conducted by Yende et al. [8]. Authors included 1799 patients discharged from emergency department with a primary diagnosis of community pneumonia. Interleukin-6 (IL-6) concentrations at hospital discharge were higher among subjects who did not survive at 100 days compared with those who survived (12.9 vs. 6.6 pg/mL, p < 0.001). This difference was not obtained among those who did and did not survive between 101 days and 1 year (7.7 vs. 6.5 pg/mL, p = NS). However, nonsurvivors compared with survivors at 1 year, nonsurvivors were older, had more co-morbid conditions, as evidenced by higher Charlson scores, and had more severe CAP on presentation, as evidenced by higher Pneumonia Severity Index and APACHE III scores.

In a recently published study by Ho et al. [6], that analyzed short-term mortality among ICU patients, a significant association between CRP concentrations at ICU discharge and subsequent in-hospital mortality was identified. In this prospective cohort study of 603 consecutive patients who survived their first ICU admission, CRP concentrations at ICU discharge were associated with subsequent in-hospital mortality in the univariate analysis (non-survivors -17.4 vs. survivors - 8.56 mg/l, p = 0.001).

In our study we were unable to reproduce these findings, since CRP was a poor prognostic marker of post-ICU mortality (AUC 0.55; 95% CI 0.42-0.67). However, in Ho et al. study [6], CRP concentrations were available only in 73% of the nonsurvivors and the number of unexpected post-ICU deaths was small (4.3%). Hence the results could be imprecise and may not be extrapolated to ICUs with higher post-ICU mortality rates.

In opposition, our data does not support the use of CRP in outcome prediction in critically ill patients, however, in comparison with Ho et al. data, our population had higher APACHE II, SOFA scores and TISS-28 as well as a larger subgroup of patients with documented infection.

In a recently published study, we also could not found a relation between CRP levels of the day of sepsis diagnosis and ICU survival [17]. Both these results demonstrate that, despite CRP has been repeatedly shown to be a sensitive marker of infection; it predicts poorly the patient outcome.

Our study has some limitations. First, this was a cohort single centre study with only 8 beds of intensive care. Second a mixed group of medical and surgical patients were included; whether CRP will have a better performance in a particular subgroup of patients, for example in patients with lower respiratory tract infection remains uncertain, but deserves further investigation. Finally severity scores were used at ICU discharge, however these scores were only developed and validated to be used in the first 24 hours after ICU admission.

Our study design had some distinctions: we analyzed separately the patients with previous documented infection as well as medical and surgical patient, and patients with end-life limitations were not excluded.

## Conclusions

Some studies suggest that persistent inflammation may precipitate deterioration in other diseases, such as cardiovascular disease, and increase long-term mortality [7]. In our data no correlation between CRP concentrations at ICU discharge and post-ICU hospital mortality could be found, with post-ICU survival appearing to be unrelated to higher levels of inflammatory biomarkers. Reasons for increased long and short - term mortality among ICU survivors are not fully understood. As a result, future studies are needed to explore the relationship between biomarkers on subsequent health-related outcomes.

## Key messages

• In the present study C-reactive protein concentrations at ICU discharge were not related to post-ICU hospital outcome. C-reactive protein despite being a sensitive marker of infection, it predicts poorly the patient outcome.

• Similar results were observed in the subgroup of ICU survivors with documented infection.

• C-reactive protein also did not demonstrate a good discriminative power of post-ICU mortality between medical and surgical patients.

## List of abbreviations

APACHE: Acute Physiology and Chronic Health Evaluation; AUC: Areas under curves; CI: Confidence intervals; CRP: C-reactive protein; ICU: Intensive Care Unit; ROC: Receiver-operating characteristic; SAPS: Simplified Acute Physiology Score; SD: Standard deviation; SOFA: Sequential Organ Failure Assessment; TISS-28: Therapeutic Intervention Scoring System-28; WCC: White cell count.

## Competing interests

The authors declare that they have no competing interests.

## Authors' contributions

All authors conceived the study. PP and LC collected the data. JS and PP drafted the manuscript. All authors helped with manuscript drafting and approved the final manuscript.

## Pre-publication history

The pre-publication history for this paper can be accessed here:

http://www.biomedcentral.com/1471-2253/10/17/prepub
